# Genetic Variation within Native Populations of Endemic Silkmoth *Antheraea assamensis* (Helfer) from Northeast India Indicates Need for *In Situ* Conservation

**DOI:** 10.1371/journal.pone.0049972

**Published:** 2012-11-21

**Authors:** Y. Tunginba Singh, Sudeshna Mazumdar-Leighton, Mahaswetta Saikia, Prashant Pant, Sochanngam Kashung, Kartik Neog, Rajen Chakravorty, Suresh Nair, Javaregowda Nagaraju, Cheerukeri Raghavendra Babu

**Affiliations:** 1 Department of Botany, University of Delhi, Delhi, India; 2 Central Muga and Eri Research and Training Institute, Ladoigarh, Assam, India; 3 International Centre for Genetic Engineering & Biotechnology, New Delhi, India; 4 Center for DNA Fingerprinting & Diagnostics, Hyderabad, Andhra Pradesh, India; 5 Center for Management of Degraded Ecosystems, University of Delhi, Delhi, India; Vetmeduni Vienna Institute of Population Genetics, Austria

## Abstract

*A. assamensis* is a phytophagous Lepidoptera from Northeast India reared on host trees of Lauraceae family for its characteristic cocoon silk. Source of these cocoons are domesticated farm stocks that crash frequently and/or wild insect populations that provide new cultures. The need to reduce dependence on wild populations for cocoons necessitates assessment of genetic diversity in cultivated and wild populations. Molecular markers based on PCR of Inter-simple sequence repeats (ISSR) and simple sequence repeats (SSR) were used with four populations of wild insects and eleven populations of cultivated insects. Wild populations had high genetic diversity estimates (H_i_ = 0.25; H_S_ = 0.28; H_E_ = 0.42) and at least one population contained private alleles. Both marker systems indicated that genetic variability within populations examined was significantly high. Among cultivated populations, insects of the Upper Assam region (H_i_ = 0.19; H_S_ = 0.18; H_E_ = 0) were genetically distinct (*F*_ST_ = 0.38 with both marker systems) from insects of Lower Assam (H_i_ = 0.24; H_S_ = 0.25; H_E_ = 0.3). Sequencing of polymorphic amplicons suggested transposition as a mechanism for maintaining genomic diversity. Implications for conservation of native populations in the wild and preserving in-farm diversity are discussed.

## Introduction

*Antheraea assamensis* is a silk-producing Lepidoptera indigenous to Northeast India and Indo-Burma [1]. Its cocoon silk has been used for centuries to weave ceremonial fabric that is golden yellow in color, lustrous and resistant to UV radiation [2], [3]. After *Antheraea yamamai,* the cocoon silk of *A. assamensis* is touted as the second most expensive silk in the world and its cultivation contributes significantly to livelihoods of indigent tribal peoples. *A. assamensis* larvae are reared outdoors, on trees of *Persea bombycina* and *Litsea monopetala* (family Lauraceae) for commercial silk production [4]. Popular practice relies on collection of wild moths from nearby forests and rejuvenations of cultivated populations. However, it is difficult to maintain cultivated stocks which crash repeatedly leading to more collections from the wild. Despite extensive efforts since 1800s, attempts to cultivate the insect outside its natural host range have failed. Varied anthropogenic developments in the area, especially felling of host trees, have led to shrinking and fragmented habitat for the insect [5]. It is thus imperative to develop tools to study natural versus cultivated populations of this economically important, endemic Lepidoptera.

**Figure 1:**
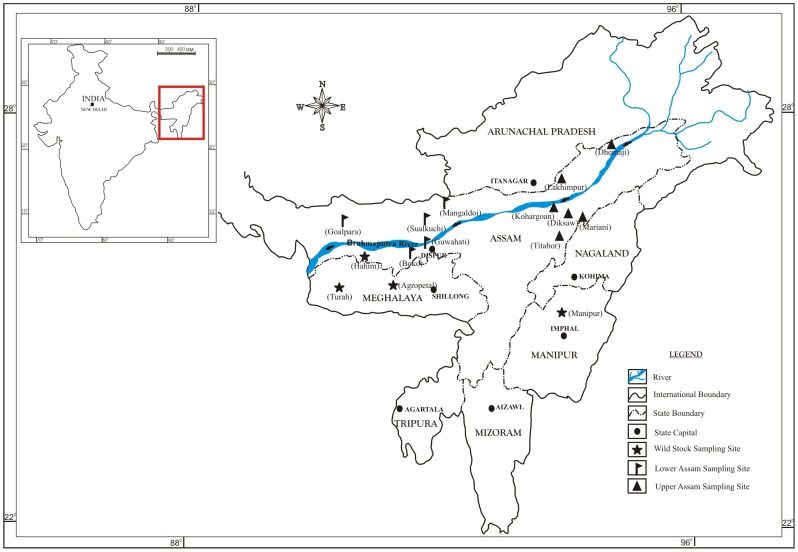
A map image showing the distribution of collection sites of *A. assamensis*. Names on the map correspond to collection sites in Table 1.

This paper investigates genetic variability in fifteen populations of *A. assamensis* moths originating from cultivated populations or wild moths from forests. Cultivated populations were collected from two regions of Northeast India, Upper Assam and Lower Assam [6], [7]. Wild populations were collected from a third topographic region, the Shillong plateau (Figure 1). Two molecular marker systems based on PCR of simple sequence repeats (SSR) and inter simple sequence repeats (ISSR) were employed. ISSR markers were developed from repeat sequences within introns of gene families available for *Antheraea* species in NCBI database [8]. SSR markers used in this study have been described previously [9]. Genetic diversity and relatedness among insect populations from this region has been examined earlier with single marker systems and smaller datasets [9], [10], [11]. SSR markers provide single-locus population data based upon sizes of amplicons obtained with unique PCR primers that flank repeat sequences. SSR markers are “co-dominant” enabling assessment of heterozygote alleles in a population [12]. Multi-locus population data based on presence/absence of amplicons is obtained using ISSR primers designed from sequence repeats [13], [14]. ISSR markers are “dominant” as they cannot distinguish heterozygote alleles, resulting in an under-representation of heterozygosity estimates [15]. Data obtained with both markers systems were evaluated for standard population genetic estimates and patterns of genetic variation among and within populations of wild and cultivated moths from different regions. Sequences and genomic organization of several ISSR amplicons were also obtained to determine the nature of genomic loci that contribute to population variations in *A. assamensis*. Results obtained are discussed for their relevance to conservation strategies.

**Table 1:** Details of collection sites (populations) of *A. assamensis* from NE India.

No.	Collection Site (Acronym)		Number of moths(M = male, F = female)	Latitude/Longitude	Altitude (m)		Geographical region
CULTIVATED POPULATIONS							
1	Boko (BK)		M = 5, F = 9	26°03’51.7″N 91°16’52.82″E	48		Lower Assam
2	Mangaldoi (MD)		M = 7, F = 9	26° 38′29.98″N 92°03’16.57″E	89		Lower Assam
3	Guwahati (NB)		M = 7, F = 6	26°09’34.62″N 91°41’27.23″E	176		Lower Assam
4	Sualkuchi (SK)		M = 10, F = 8	26°31’10.61″N 91°30’42.66″E	71		Lower Assam
5	Goalpara (GP)		M = 12, F = 4	26°17’54.49″N 90°32’28.82″E	44		Lower Assam
6	Mariani (MR)		M = 2, F = 16	26°45’06.07″N 94°24’43.02″E	120		Upper Assam
7	Dhemaji (DM)		M = 3, F = 10	27° 39′49.08″N 94°55’41.02″E	215		Upper Assam
8	Diksaw (D)		M = 10, F = 10	26°46’35.73″N 94°07’23.42″E	96		Upper Assam
9	Kohargaon (KG)		M = 12, F = 6	26°45’29.59″N 93°58’00.43″E	86		Upper Assam
10	Titabor (TB)		M = 7, F = 13	26°16’13.44″N 93°51’18.96″E	142		Upper Assam
11	Lakhimpur (LK)		M = 8, F = 7	27°13’54.10″N 94°05’24.33″E	92		Upper Assam
WILD POPULATIONS							
12	Hahim (HA)	M = 15, F = 6		25°54’20.65″N 91°03’29.02″E	331	Shillong Plateau	
13	Manipur (MN)	M = 6, F = 12		25°16’07.48″N 93°56’26.99″E	1941	Shillong Plateau	
14	Turah (TR)	M = 14, F = 6		25°41’35.54″N 90°26’23.99″E	464	Shillong Plateau	
15	Agropetal (AG)	M = 10 F = 12		25°41’35.54″N 90°26’23.99″E	464	Shillong Plateau	

## Results

### Genetic Diversity Estimates for A. assamensis Populations Using SSR and ISSR Markers

High genetic diversity estimates were obtained for the full data set comprising of fifteen populations (Table 1) using both marker systems. Table 2 and 3 showed that Nei’s gene diversity (H_i_ = 0.37±0.02) estimated for all populations using all ISSR primers was similar to Nei’s expected heterozygosity value (H_E_ = 0.37±0.25) obtained from SSR data. Both ISSR and SSR primers yielded variable numbers and sizes of amplicons. Bayesian heterozygosity estimates (H_S_) obtained with all ISSR primers was more circumspect than H_i._ However, trends observed with the frequentist (H_i_) and Bayesian estimates (H_S_) were consistent. For instance, AhISSR5 showed lowest percentage polymorphism, lowest H_i_ and H_S_ values among all the ISSR primers tested (Table 2). An excess of homozygotes was observed with each SSR primer (Table 3). Table 4 shows that several cultivated populations from Upper Assam region had low estimates of genetic diversity (H_i_ and H_S_). Nei’s heterozygosity estimates could not be estimated for insect populations from this region, as a single allele (monomorphic locus) was amplified with each SSR primer tested. A wild population from the Shillong plateau (Agropetal) showed the highest percent polymorphism, highest number of haplotypes, H_i_ and H_T_ estimates using both marker systems (Table 4). Two private alleles were detected in this population with Ahsat020 primer (not shown). Populations with highest H_S_ values were Hahim (a wild population from the Shillong plateau) and Mangaldoi (a cultivated population from Lower Assam).

**Table 2:** A primer-wise comparison of all *A. assamensis* populations for percentage polymorphism (%P), Nei’s gene diversity (H_i_), and Bayes heterozygosity estimate (H_S)_.

ISSR Primer	Sequence	No. of amplicons (% P)	Nei’s H_i_	Bayes H_S_	Source & nature of amplicons cloned	GenBank Accession Numbers
AhISSR2	(GAA)_6_C	6 (100)	0.3±0.03	0.29±0.02	NB-9_1316P_, SK-7_684M,_ SK-7_643M_	GQ273748, EU123529, EU123530
AhISSR4	(TCG)_ 6_C	7 (100)	0.3±0.04	0.25±0.02	NB-11_893M_	GQ273750
AhISSR5	(CGA)_ 6_T	4 (75)	0.28±0.06	0.18±0.02	NB-5_437P_, TR-4_547M_, MD-8_547M,_ DM-1_547M_	GQ273749, GQ273755, GQ273756, GQ273757
AhISSR11	C(GATA)_ 5_	8 (100)	0.3±0.02	0.2±0.01	GP-16_609M_, GP-16_825M_	GQ273753, GQ273754
AhISSR12	(GATA)_ 5_T	11 (100)	0.39±0.02	0.28±0.01	MN-1_1064M_	GQ273752
AhISSR13	(GATA)_ 5_A	6 (100)	0.37±0.01	0.28±0.01	GP-13_1249P_, GP-14_607M_	EU123527, EU123528
AhISSR110	(GATA)_ 5_C	8 (100)	0.39±0.02	0.28±0.01	TR-4_1456P_, TR-4_1452P_, HA-3_1400P_	DQ872512, DQ872513, GQ273751
All primers			0.37±0.02	0.24±0.01		

GenBank Accessions for cloned and sequenced amplicons are provided. The population and moth from which the amplicon was obtained are indicated in uppercase. Amplicon size and nature of the locus is shown in subscript. The subscript “P” refers to a polymorphic locus (amplicon present in some individuals of a particular population), while the subscript “M” refers to a monomorphic locus (amplicon present in all individuals of that population).

**Table 3:** Estimates of heterozygosity calculated from SSR data. N_a_ is observed number of alleles (amplicons produced) and N_e_ is the effective number of alleles; H_E_ and H_O_ refer to expected and observed heterozygosity values; Asterisk (*) denotes significant deviation from HWE at p<0.05.

SSR Primers	Repeat motif	N_a_	N_e_	H_E_	H_O_
AaSat020F	(TCGTG)_5_	5	1.1	0.13	0.06*
AaSat044	(AT)_20_	3	1.38	0.38	0.24*
AaSat065F	(AT)_11_N_n_(AT)_11_	4	2.16	0.62	0.47*
All primers			1.5±0.5	0.37±0.25	0.26*±0.2

**Table 4:** Population-wise comparison of percentage polymorphism (%P), Nei’s gene diversity (H_i_) and Bayesian heterozygosity (H_S_) values determined from ISSR-PCR data and Nei’s average gene heterozygosity (H_T_) values determined from SSR-PCR data. n.d. = not detectable. Hap: Number of haplotypes.

Populations	ISSR			SSR		
	% (P)	Nei’s H_i_	Bayesian H_S_	Hap		H_T_
CULTIVATED POPULATIONS (*Lower Assam Region*)						
Boko	80	0.29±0.19	0.29±0.01	6	0.25	
Mangaldoi*	82	0.32±0.18	**0.31±0.02**	9	0.28	
Guwahati*	78	0.31±0.25	0.30±0.02	6	0.32	
Sualkuchi*	52	0.20±0.21	0.22±0.01	5	0.20	
Goalpara*	54	0.19±0.20	0.21±0.02	5	0.26	
CULTIVATED POPULATIONS (*Upper Assam Region*)						
Mariani	52	0.18±0.20	0.19±0.02	1	n. d.	
Dhemaji*	58	0.25±0.23	0.28±0.02	1	n. d.	
Diksaw	64	0.22±0.19	0.21±0.01	1	n. d.	
Kohargaon	44	0.15±0.20	0.21±0.02	1	n. d.	
**Titabor**	**30**	**0.09±0.17**	**0.14±0.01**	**1**	**n. d.**	
Lakhimpur	40	0.14±0.20	0.17±0.02	1	n. d.	
WILD POPULATIONS (*Shillong Plateau Region*)						
Hahim*	64	0.35±0.18	**0.31±0.01**	7	0.36	
Manipur*	58	0.23±0.22	0.27±0.02	4	0.29	
Turah*	78	0.27±0.20	0.26±0.02	8	0.36	
**Agropetal**	**90**	**0.38±0.16**	**0.26±0.02**	**13**	**0.54**	

Asterisks (*) indicate populations from which selected loci were cloned and sequenced. Populations with highest and lowest genetic diversity estimates are shown in bold.

### Partitioning Genetic Variation among and within Populations Using ISSR and SSR Markers

AMOVA showed that majority of genetic variation in the full data set detected by both markers was found within the fifteen populations (64% with ISSR markers, F_ST_ = 0.36 and 82% with SSR markers, F_ST_ = 0.18 at P-value = 0, Table S1). Comparison of the group of eleven cultivated populations versus the group of four wild populations using ISSR marker data (Table 5A) indicated that while most of genetic variation (57.54%) resided within the populations examined, 17.94% genetic variation was found between the two groups, F_CT_ = 0.18. Results with SSR marker data (Table 5C) showed that most of the genetic variation (79.58%) was found within cultivated versus wild populations, but the genetic variation among these groups was only 5.1%, F_CT_ = 0.05. When all populations were grouped into three according to region (cultivated populations from Upper Assam region, cultivated populations from Lower Assam region; wild populations from Shillong plateau region), approximately 20% of the total genetic variation (F_CT_ = 0.2) was observed between the three groups with both markers (Table 5B1, 5D1). Interestingly, when the two groups of cultivated populations from Upper Assam and Lower Assam regions were compared amongst themselves, approximately 62% within-population genetic variation and F_ST_ of 0.38 was observed with both markers (Table 5B2, 5D2). However, the two markers yielded very different results for partitioning of genetic variation among populations within the two groups of cultivated populations. The F_SC_ value was 0.26 with ISSR data (Table 5B2) but only 0.004 with SSR data (Table 5D2).

**Table 5:** Partitioning by AMOVA of genetic variation in *A. assamensis* moths from (A) cultivated and wild populations, (B1) three regions of Northeast India and (B2) cultivated populations from 2 regions of Northeast India using ISSR marker data; (C) cultivated and wild populations, (D1) three regions of Northeast India and (D2) cultivated populations from 2 regions of Northeast India using SSR marker data.

	Source of variation	d.f.	Variance components	Percentage of variation	Fixation indices	P-value*
A.	Among groups	1	1.6	17.94	F_CT_ = 0.18	0.00196±0.00136
	Among populations within groups	13	2.18	24.52	F_SC_ = 0.3	0
	Within populations	192	5.12	*57.54*	F_ST_ = 0.43	0
	Total	206	8.9			
B1.	Among groups	2	1.72	20.28	F_CT_ = 0.20	0
	Among populations within groups	12	1.64	19.33	F_SC_ = 0.24	0
	Within populations	192	5.12	*60.39*	F_ST_ = 0.40	0
	Total	206	8.48			
B2.	Among groups	1	1.27	16.65	F_CT_ = 0.17	0.0001±0.00009
	Among populations within groups	9	1.67	21.77	F_SC_ = 0.26	0
	Within populations	140	4.71	*61.58*	F_ST_ = 0.38	0
	Total	150	7.65			
C.	Among groups	1	0.02	5.09	F_CT_ = 0.05	0.11632±0.00964
	Among populations within groups	13	0.07	15.33	F_SC_ = 0.16	0
	Within populations	363	0.37	*79.58*	F_ST_ = 0.20	0
	Total	377	0.47			
D1.	Among groups	2	0.10	19.97	F_CT_ = 0.2	0.001±0.001
	Among populations within groups	12	0.01	2.91	F_SC_ = 0.04	0.008±0.003
	Within populations	363	0.37	*77.12*	F_ST_ = 0.23	0
	Total	377	0.48			
D2.	Among groups	1	0.15	37.82	F_CT_ = 0.38	0
	Among populations within groups	9	.001	0.27	F_SC_ = 0.004	0.336±0.015
	Within populations	233	0.25	*61.9*	F_ST_ = 0.38	0.003±0.002
	Total	243	0.4			

* Significance tests were performed for all P-value estimates at 1023 permutations.

### Genetic Structure of Wild and Cultivated *A. assamensis* Populations from Different Regions

**Figure 2:**
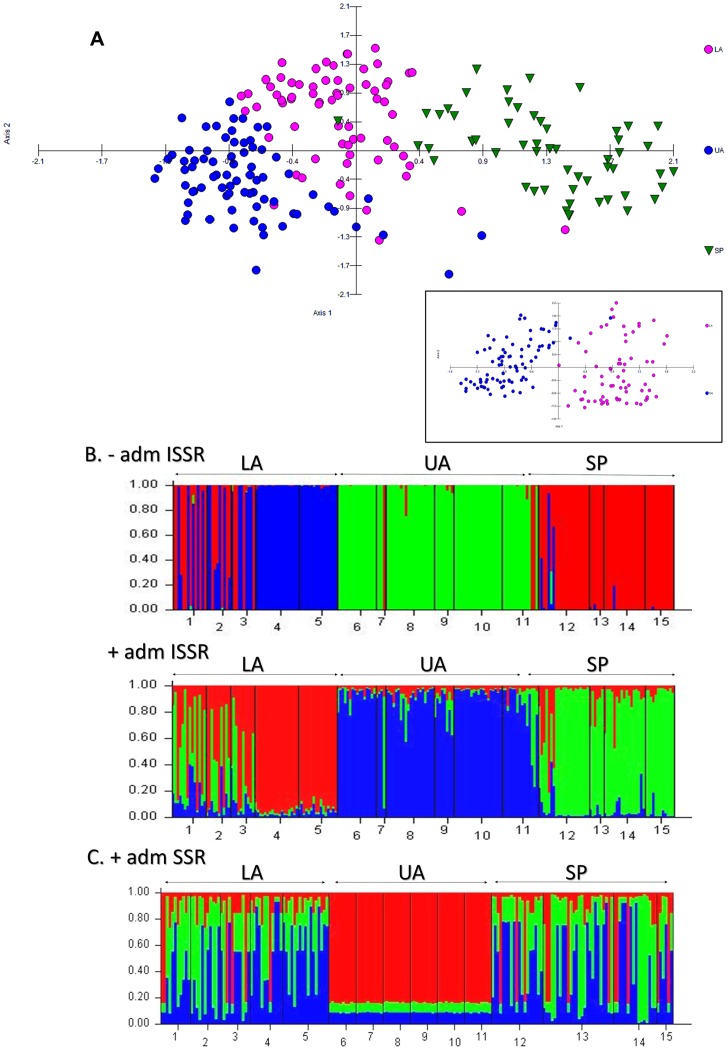
Analyses of sampled populations using PCO and STRUCTURE. Genetic relationships were determined using (A) PCO analyses of 207 ISSR fingerprints obtained for fifteen populations collected from 3 regions. Designations for regions are: Upper Assam or UA (cultivated populations), Lower Assam or LA (cultivated populations) and Shillong plateau or SP (wild populations). Inset shows PCO analyses of ISSR fingerprints from eleven cultivated populations from Upper Assam and Lower Assam regions. Putative population genetic structure (B) was obtained with Admixture (+adm ISSR) and without Admixture (-adm ISSR) settings at K = 3 using ISSR data with STRUCTURE [59]. Each vertical bar represents a moth distributed into 1 of K colored clusters. Populations 1 (BK), 2 (MD), 3(NB), 4 (GP) and 5 (SK) are cultivated populations from Lower Assam (LA); populations 6 (MR), 7 (DM), 8 (D), 9 (KG), 10 (TB) and 11 (LK) are cultivated populations from Upper Assam (UA); populations 12 (HA), 13 (MN), 14 (TR) and 15 (AG) are wild populations from Shillong plateau (SP). Population acronyms are expanded in Table 1. Putative population genetic structure (C) was obtained with Admixture (+adm SSR) setting at K = 3 for 15 populations (189 insects) using SSR data as above.

PCO analyses using Euclidean distances among 207 ISSR fingerprints for cultivated and wild populations from different regions (Figure 2) accounted for 17% and 10% of the total genetic variance on principal axis 1 and principal axis 2, respectively. Figure 2 also shows that while cultivated insects from Upper Assam were mostly distinct from wild insects of the Shillong Plateau, some insects from Lower Assam were similar to wild insects suggesting limited mixing/gene flow. Figure 2 inset shows results of PCO analyses comparing cultivated populations from two regions, Upper Assam and Lower Assam where the first principal component axis explained 14% of the genetic variance between the two groups. This result was supported by previous pair-wise estimates of Nei’s genetic distance obtained from ISSR marker data comprising of fifty loci (Table S2). Among hierarchical clustering methods, UPGMA provided the best fit for ISSR data with fifty loci and indicated clustering of populations based upon source (wild and cultivated) as well as region (Figure S1). A standardized Mantel statistic of r = 0.4675 was obtained for the association between geographic and Nei’s genetic distances with P = 0.001 (for 999 replicated randomizations). This correlation suggested that the some of the populations differentiated *in situ* rather than having differentiated elsewhere and migrating to their current locations.

In order to further explore the results described above, a Bayesian approach for clustering related individuals into discrete units (or K), was applied. For ISSR data with 50 loci, and K = 3, discrete groups of populations comprising wild insects from Shillong Plateau region, cultivated insects from Upper Assam and Lower Assam regions were retrieved with both admixture and no-admixture models (Figure 2B). Results from the admixture model showed “mixing” among three cultivated populations of Lower Assam, i.e. Boko, Guwahati and Mangaldoi with insects from wild populations (Figure 2B). It is unclear if the observed admixing was due to migration or anthropogenic introduction of wild insects into cultivated populations. Two cultivated populations from Lower Assam (Goalpara and Sualkuchi) were discrete. Application of SSR data gave the most cohesive results at K = 2 and K = 3 (Figure 2C), where cultivated insects of Upper Assam were distinct from all other insects.

### Genomic Variability in Populations of *A. assamensis* and Motifs of Transposable Elements

Several ISSR amplicons were cloned into pDrive vector and their sequences submitted to GenBank (Table 2). These sequenced products originated from amplicons from loci that were (i) monomorphic (NB11_893M_) for Upper Assam (UA), Lower Assam (LA) and Shillong plateau regions (SP); (ii) polymorphic for LA and SP but absent from UA (NB5_437P_); (iii) polymorphic (TR4_1452P,_ TR4_1456P,_ HA3_1450P_) for UA, LA and SP; (iv) polymorphic (GP13_1249P_) and monomorphic (GP14_607M_) for moths of the same population; and (v) polymorphic (NB9_1316P_) and monomorphic (SK7_684M_, SK7_643M_) for moths from different populations. Amplicons representing loci that were (i) monomorphic from the same moth of a population (GP16_825M_, GP16_609M_) and (ii) monomorphic (MD8_547M_ of LA, TR4_547M_ of SP and DM1_547M_) were also analyzed to determine if such monomorphic loci contain the same sequence. About 10 kbp of the *A. assamensis* genome was sequenced. Sequence analyses of these cloned loci revealed that without exception, primers based on the same repeat sequence but containing different mononucleotide anchor amplified unique, non-redundant loci across the *A. assamensis* genome.

**Figure 3:**
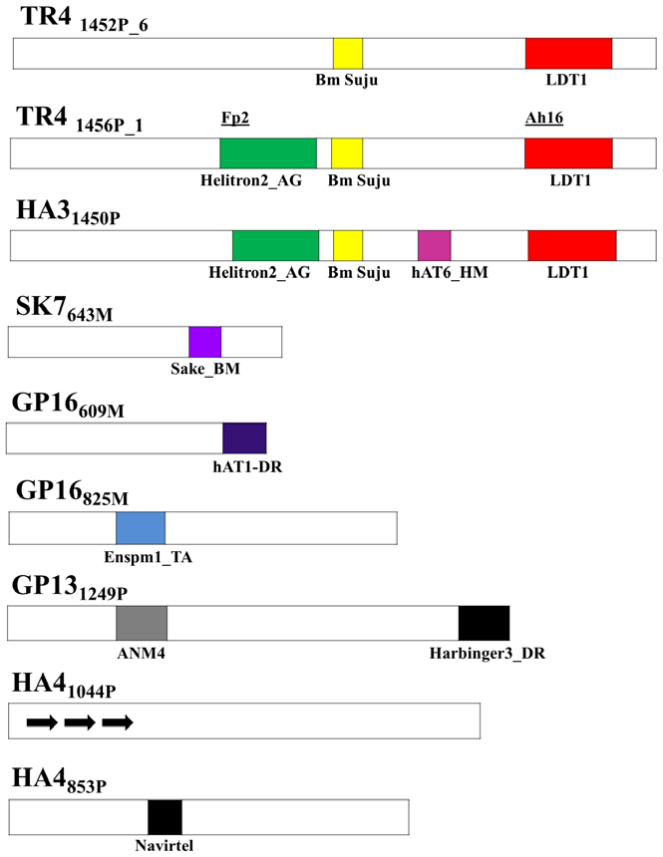
A map showing positions of motifs resembling putative transposable elements (boxes shown in different colors) within sequenced ISSR amplicons. Abbreviations are as described in Table 2. Arrows denote tandem repeats. The bold lines ( ) indicate position of nested primers (Fp2 and Ah16) within amplicon EU872512.

Results obtained after analyzing the data with Spectral Repeat Finder (SRF) indicated the presence of interspersed, imperfect repeats ranging from 2 to 10-mers with a low spectral frequency of.0005 to.001. The TR4_1456P_ locus contained the largest copy number (103) of such a repeat. Amplicons obtained with tri-nucleotide repeat primers had 40%GC compared with primers based on tetra-nucleotide repeat sequence (34%GC). Most sequences were AT-rich and contained stop codons in all three frames especially at the 5′ end. Sequence of the TR4_1456P_ amplicon contained twenty copies of a TATAAAAT sequence while MN1_1064M_ locus contained a 40 bp AT-rich region. No tandem, perfect simple sequence repeats were observed in any amplicon using either SRF or mREP softwares. Analyses with RepeatMasker and Consensus softwares (Figure 3) revealed the occurrence of a 109 bp sequence resembling the SAKE (L2) LINE element from *B. mori* in SK7_643M_ loci; a 55 bp sequence resembling EnSpm DNA transposable element from *Triticum aestivum* in GP16_609M_; a 57 bp sequence resembling an *A. mylitta* transposase psuedogene Anm4 in GP13_1249P;_ a 50 bp sequence resembling a Helitron from *Anopheles gambiae* and a 166 bp sequence resembling a non-LTR retrotransposon, Jockey from the Lepidoptera, *Lymantria dispar* in TR4_1456P_, TR4_1452P_ and HA3_1450P_ loci. A simple sequence repeat (CTAAG)_n_ was identified in TR4_1456P_ and TR4_1452P_ close to the Jockey-like stretch. About 50% of the ISSR amplicons sequenced in this study were associated with remnants/footprints of transposable elements. Furthermore, the (GATA)_n_ primer binding regions along with an additional 12 to 28 bp were found to resemble hAT and the Harbinger elements from zebra fish in the sequences of GP16_825M_ and GP13_1249P_ loci. During searches of the GenBank databases, the (GATA)_n_ primer binding sequences along with 6–12 bases were found to resemble micro-satellite regions from a wide variety of organisms. BLAST hits of the EST databases often indicated that the 3′ ends of the amplicons contained regions of coding sequences suggesting that intron-exon junctions may be spanned during amplification with these ISSR primers. This may explain an observation made earlier that the stop codons were generally clustered in the 5′ends of the sequences. In the case of GP13_1249P_ and SK7_643P_ sequences obtained with primer AhISSR13 and AhISSR5, a 130 bp sequence was common in both amplicons immediately proximal to the forward primer sequence. In fact, several sequenced amplicons obtained with the same primers contained perfectly conserved sequences immediately proximal to the forward primers, suggesting the occurrence of multiple copies of repetitive regions in the *A. assamensis* genomes. Finally, we observed identical sequences within a monomorphic locus of 547 bp amplified from several individuals belonging to the three population clusters. This result supported a basic premise of ISSR-PCR that shared bands (scored as 1) may represent homologous loci.

### Southern Blot with a Repetitive Element from a Polymorphic Locus as Probe Hybridizes to Lepidopteran Genomes and is Organized Differently in Populations of *A. assamensis*

**Figure 4:**
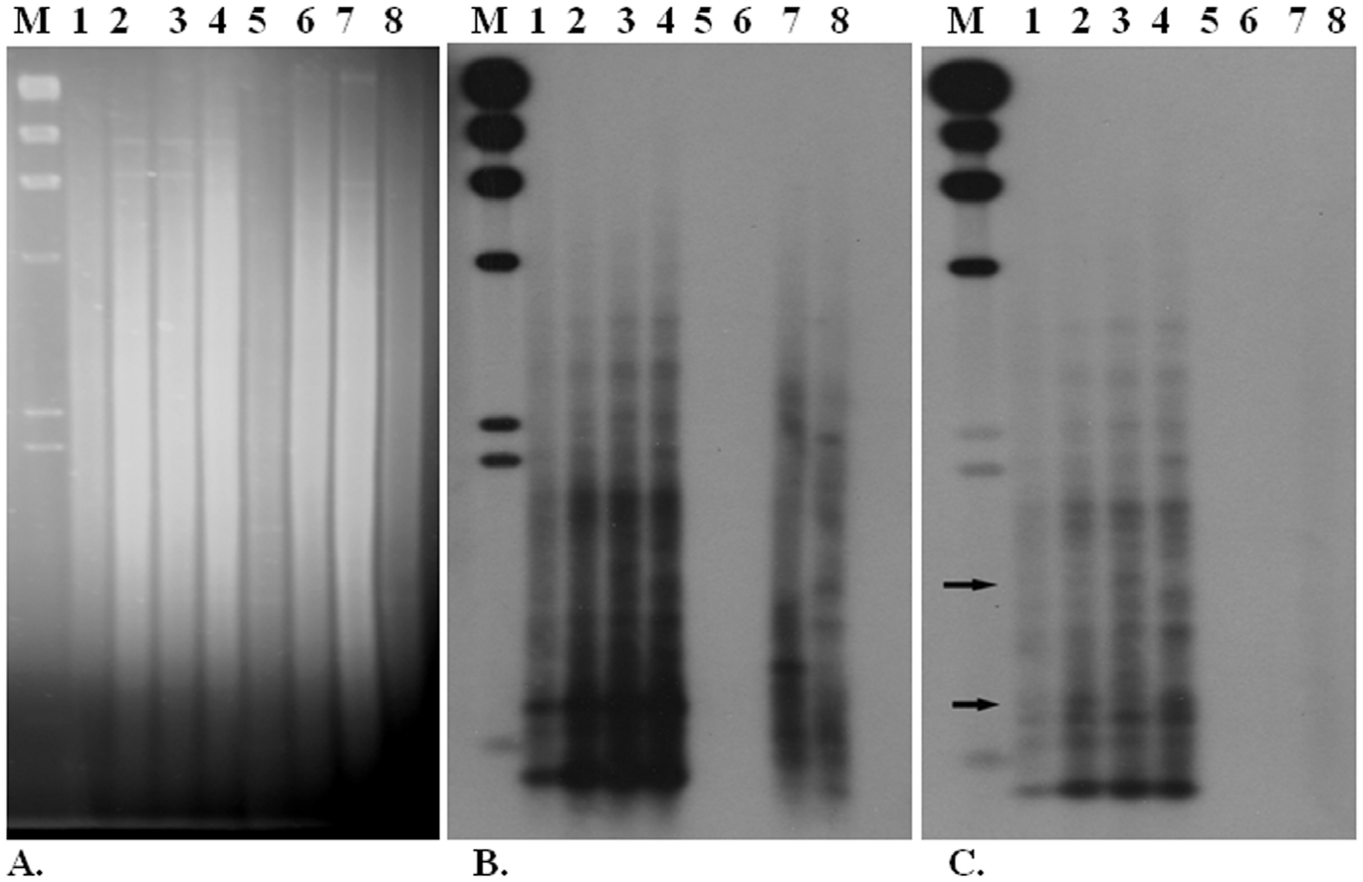
Southern hybridization of various silkmoths using a putative transposable element amplicon from *A. assamensis* as probe. Genomic DNAs (A) of *A. assamensis moths* BK12 (lane 1), TR3 (lane 2), TR4 (lane 3), KG8 (lane 4); *B. mori* (lane5), *S. cynthia* (lane 6), *A. mylitta* (lane 7) and *A. proylei* (lane 8) were digested with *Hae* III. Southern hybridization of the digested DNAs with an 800 bp amplicon containing a putative mariner-like element are shown after (B) a low stringency wash and (C) a high stringency wash. Lane M shows λ DNA restricted with *Hin*d III as a size marker. Arrows denote hybridization signals that distinguish moths from different populations.

An amplicon of 800 bp with sequence similarity to lepidopteran transposable elements was obtained by nested amplification from the TR4_1456P_ locus (Figure 3) and used as a probe in Southern hybridization with *Hin*d III and *Hae* III digested DNA from *A. assamensis*, *A. proylei, A. mylitta, B. mori* and *S. cynthia* (Figure 4A). All four individuals of *A. assamensis* examined (TR3 and TR4 from Turah of SP, BK12 from Boko of LA and KG8 from Kohargaon of UA) contained the 1456 bp amplicon. Low stringency salt washes showed hybridization of the probe with heterologous sequences from *A. proylei, A. mylitta* but not *B. mori* and *S. cynthia* indicating that the probe was specific for *Antheraea* species (Figure 4B). PCR amplification with Fp2/Ah16 primers (used to generate the probe) also supported this result as no amplification products were obtained with *B. mori* and *S. cynthia* (not shown). High stringency washes revealed that the probe (with an internal restriction site for *Hae* III) hybridized to multiple but distinct loci in the genomes of individual *A. assamensis* that came from different populations (Figure 4C). These results suggested that these repetitive elements occurred in multiple copies, were interspersed in the *Antheraea* genomes and distributed differently in moths of different populations. Nei’s genetic distances between Boko (a cultivated population from Lower Assam region) and Kohargaon (a cultivated population from Upper Assam region) was 0.1644, Boko and Turah (a wild population from the Shillong plateau region) was 0.1839; Turah and Kohargaon was 0.3011 (Table S2).

## Discussion

### High Genetic Diversity Detected in *A. assamensis* Using SSR and ISSR Markers

Cocoon silk of *A. assamensis* is a major cottage industry of economic and cultural significance in North-eastern India [16]. Rearing of cultivated populations depends on re-invigoration by insects collected from wild populations. Combined use of two marker systems with same set of insect populations can improve the reliability of information obtained for devising robust conservation strategies. Estimated gene diversity indices in *A. assamensis* ISSR markers were high at 0.37±0.02 (H_i_) using frequentist methods and 0.24±0.01 (H_S_) while using Bayesian principles (Table 2). Even though actual values differed, trends between estimates obtained with frequentist approach (H_i_) and Bayesian approach (H_S_) were similar. Similar discrepancies in numerical values have been observed earlier and attributed to the different approaches for these measures [17]. Expected heterozygosity (H_E_) estimated from SSR data was also high at 0.37±0.23 (Table 3). Similar high estimates of gene diversity have been reported for smaller data sets of *A. assamensis* using molecular markers [9], [10], [11], suggesting that intra-specific diversity is high and the insect germplasm has sufficient genetic potential to envisage well-designed conservation efforts. Average heterozygosity (H_O_) observed in Lepidoptera using allozyme analysis is 0.127 [18], ranging from a low of 0 for *E. mana* to a high of 0.46 for *O. brumata* [30/19]. Results from this study (Table 3) resembled previous report where the observed heterozygosity with SSR markers was usually lower than expected due to null alleles and/or Wahlund’s effect [9]. High genetic diversity, percent polymorphism and low gene flow have been reported for silk-producing insects cultivated in India, such as Indian Eri silk moth, *Samia cynthia;* Indian Tussah silk moth, *Antheraea mylitta* and races of *Bombyx mori* [20]–[26].

### High Genetic Diversity in Wild Populations, Cultivated Populations from Different Regions Differ in Diversity Estimates

High gene diversity estimates for wild populations from the Shillong plateau with both marker systems (Hi = 0.25±0.15; Hs = 0.28±0.01 with ISSR markers and H_E_ = 0.42±0.25 with SSR markers) indicated the need to preserve forests in North-east India as repositories of diverse germplasm of *A. assamensis*. The highest number of haplotypes (12), and two private SSR alleles was detected in the Agropetal population which contained native insects. Similar reports have been reported earlier for wild populations from this region using single markers systems [9], [10], [11]. Genetic diversity estimates observed in all cultivated populations were H_i_ = 0.24±0.17; H_S_ = 0.21±0.01 for ISSR markers and H_E_ = 0.21±0.11 with SSR markers. While several observations for cultivated populations held between the two marker systems, some did not. For example, in contrast to the ISSR data, cultivated populations of Upper Assam were monomorphic with all eleven SSR markers (Table 4). Nevertheless, both genetic marker systems clearly indicated that cultivated populations from Upper Assam were the most homogenous and had lowest estimates of genetic diversity (H_i_ = 0.19±0.17; H_S_ = 0.18±0.01; H_E_ = 0). Both Nei’s H_i_ and Bayesian H_S_ values were the lowest for Titabor (a cultivated population from Upper Assam) which also had the lowest percentage polymorphism and least number of polymorphic bands (Table 4). Interestingly, in a report with 6 populations of *S. cynthia* collected from sites similar to our study, moths from Titabor also showed the lowest genetic heterozygosity values [25]. The significance of this observation is unclear but could refer to local rearing practices promoting inbreeding or reduced gene flow from other populations. In contrast to Upper Assam, cultivated populations from Lower Assam had higher genetic diversity estimates in this study (H_i_ = 0.24±0.16, H_S_ = 0.25±0.01 with ISSR markers and H_E_ = 0.3±0.21 with SSR markers). Reasons for these observations are unclear, but could be recent introduction of wild parents to invigorate cultivated populations of Lower Assam. The values from Upper Assam populations could be a snapshot of genetic diversity values in populations containing more inbred insects, prior to a stock invigoration. More work is needed to understand and monitor the genetic consequences of introducing wild insects into cultivated populations.

### Genetic Variation is High within Wild and Cultivated Insect Populations from Different Regions

Both molecular marker systems indicated that genetic variation was partitioned within individuals of the fifteen populations rather than among groups of populations based upon topography or origin (Table 5). The import of high within-population genetic variation is unclear. It could represent a genetic strategy of an endemic insect species to survive in its habitat. Occurrence of high genetic variation within populations is of particular significance in developing conservation strategies for animals to survive under changing environmental conditions or biotic stress [27]. It can also be of significance for development of pure breeding lines at different locations [28]. Comparison of patterns of genetic variation among the group of wild populations and the group of all cultivated populations is interesting, as this distinction likely underlines the use of insects from wild populations to invigorate cultivated populations. Interestingly, a F_CT_ = 0.18 value was observed among these two groups with ISSR markers (Table 5A) and a low F_CT_ = 0.05 was observed with SSR markers (Table 5C). The SSR markers gave better results for among groups of populations based on region/topography. F_CT_ values were similar for both markers (0.2) when three groups of populations from different regions (wild populations from Shillong plateau, cultivated populations from Lower Assam and cultivated populations from Upper Assam) were examined (Table 5B1, 5D1). Topography and associated factors may thus be a factor influencing patterns of genetic variation observed in this insect. Genetic variation observed among and within cultivated populations from Upper Assam region versus Lower Assam region (Table 5B2, 5D2) could be influenced by low genetic diversity estimates and preponderance of monomorphic loci in Upper Assam populations (Table 4). Reasons for distinctions between cultivated populations of Upper and Lower Assam regions remain, but do not include choice of host plant species. All larvae of cultivated populations examined in this study were reared on natural stands of *P. bombycina,* a highly out-bred species. Further work is needed to investigate the role of host plant genotype (if any) on population genetics of cultivated insect populations.

### Population Structure of Wild and Cultivated Populations of *A. assamensis* from Different Regions

Principal coordinate analyses (Figure 2) and AMOVA (Table 5) indicated that insects of wild populations from the Shillong Plateau were distinct from insects of almost all cultivated populations from Upper Assam. Insects of the cultivated populations from Upper Assam were distinct from those of Lower Assam. While these results suggested that both source of the insects (i.e. wild versus cultivated) and regional topography were possible factors influencing genetic variation observed between populations of *A. assamensis,* some mixing of genotypes especially between wild populations and the geographically proximal cultivated populations of Lower Assam was also indicated. Moderate genetic distance estimates (Table S5) and its concurrence with geographic distance among populations observed in this study may be attributed to low migration and mixing of the genotypes on a limited geographical scale. The observation that genetic differentiation increased with increasing geographic distance further supports the contention that the wild moths do not migrate far [11], [29], [30]. Analysis with STRUCTURE using ISSR data indicated a putative population structure where wild populations and cultivated populations from Upper Assam and Lower Assam formed discrete clusters (Figure 2B). Populations of Lower Assam were further organized into at least two sub-clusters, one of which showed mixing with wild populations due to migration and/or anthropogenic intervention. Limited migration has been reported among wild populations of the insect [11]. In this study, SSR data could not be used to distinguish wild populations from cultivated populations from Lower Assam (Figure 2C), reflecting AMOVA results (low F_CT_ = 0.05, between cultivated and wild populations) observed earlier (Table 5C).

In contrast with the genetic data, little geographic structure was observed with phenotypic data analyzed for the fifteen populations in this study. Sexual dimorphism for selected traits (moth weight, cocoon weight and wing-length) was evident (Figure S2). Positive correlations were observed between traits such as moth weight and shell weight, an economic trait (Figure S3). In a 2-way (sex and locality) ANOVA of the data, the interaction was not statistically significant, indicating that sex-specific differences were not locality-related. However, attempts to hierarchically cluster the populations failed, suggesting that the morphological traits considered here were not sufficiently distinct to group the insects based upon source or region. Sexual dimorphism and intra-specific variation in venation patterns and vein-thickness of *A. assamensis* moths has been reported recently [31]. Our results suggest that measured phenotypic traits may be under stabilizing selection with similar optima in all populations, since the low rate of migration would be sufficient to allow the populations to diverge by selection or genetic drift if stabilizing selection were not the same at all locations. The nature of this stabilizing selection is a matter of conjecture and could well be constraints imposed by a common life history [32]. Alternatively, factors such as reduced gene flow between some populations could be responsible.

### Sequence Analyses of Polymorphic Loci in *A. assamensis* and Southern Hybridization Reveal Motifs from Several Transposable Elements

Amplicons representing several polymorphic and monomorphic loci from moths within the same and different populations were sequenced (Table 2). About 50% of the sequenced amplicons representing polymorphic loci contained regions ranging from 28 to 166 bp with high sequence similarity to known class II transposable elements (Figure 3). In the case of TR4_1456P_ amplicon, sequence motifs of more than one mobile genetic element were detected. The association of transposable elements with population genetic variation is well known for animals and plants [33], [34], [35]. Most of these studies cite the occurrence of transposable elements and remnants of transposition events in introns consistent with our design of intron repeat-based ISSR primers yielding amplicons containing putative transposable elements (Table S3). Associations of simple sequence micro-satellite repeats (as used for design of ISSR primers) with transposable elements have been reported in other organisms such as rice [36], [37]. Transposable elements like *Bilbo* have been shown to maintain genetic variability within populations and influence population structure of *Drosophila* species [38], [39]. In *D. melanogaster*, transposable elements (some of which occur in introns) which show population heterogeneity have been attributed a major role in adaptation to local temperate climates [40]. Transposable elements are known to constitute 35% of the *Bombyx mori* genome [41]. Thus, they may be strongly implicated in population genetic variations and evolution of Lepidoptera. Southern hybridization studies were carried out with DNA from five sericigenous Lepidoptera with a 800 bp probe containing a 166 bp region resembling a non-LTR retrotransposon Jockey element from *Lymantria dispar.* The probe hybridized with DNA from *A. assamensis, A. mylitta* and *A. proylei* but not *B. mori* or *S. cynthia*, suggesting that it was specific for *Antheraea* species. In *A. assamensis*, the probe bound to multiple but identical loci within *Hin*d III digested DNAs of four moths from different populations (not shown). In the case of *Hae* III digested DNAs of the same four *A. assamensis* moths, the probe hybridized to multiple loci, some of which were distinct for moths from different populations (Figure 4) suggesting that sequences contained in the probe might be involved in origin of polymorphic loci in *A. assamensis*. The probe was a fragment of an amplicon obtained with AhISSR 110 primer whose sequence was based on a (GATA)_n_ repeat. Our Southern hybridization result is reminiscent of the autosomal, non-allelic hypervariable Bkm DNA loci in the moth *Ephestia kuehniella* known to be associated with mobile genetic elements [42]. Insertion of class II transposable element belonging to Helitron-like superfamily has recently been shown to interrupt micro-satellite loci in *Ostrinia nubilalis, Pectinophora gossypiella* and *B. mori* [43]. Further research is required to investigate the nature and roles of transposable elements in Antheriids as they may have be relevant for breeding experiments and the ability of progeny of diverse populations to adapt and survive in a particular region. Local micro-climate and disease/predator loads in localities from where the collections for this study may be variable and pose unique challenges to the survival of the species, especially for native populations.

In conclusion, the use of two molecular markers as described in this study strongly indicates that wild populations need to be preserved in their natural habitats as invaluable germplasm for invigorating cultivated populations. Our results coincide with other reports based on single marker systems [9], [10], [11] which provide snapshots of allele frequencies in different populations of *A. assamensis*. Further research is necessary to understand the role of transposable elements and other genomic mechanism(s) that can influence genetic variability within populations of *A. assamensis.* Taken together, the importance of monitoring the history and genetic consequences of introduction of wild moths (mixing) during invigoration of cultivated stocks is paramount. Molecular markers can be useful for such efforts. Both wild and cultivated populations of *A. assamensis* are endemic and restricted to Northeast India [16]. If considered as a meta-population, then dynamic strategies and different priorities may be necessary for conservation of native insects and management of cultivated insects.

## Materials and Methods

### Collection Sites and Morphometric Measurements of Insects

Cocoons of *A. assamensis* were randomly collected from forests, silk farmers, state, and central government insect rearing depots in Northeast India. To the best of our knowledge, no collection was made from a restricted or protected area. A total of 256 cocoons were collected representing fifteen collection sites (henceforth referred to as populations). The location, altitude and latitude of these fifteen sites were ascertained from governmental maps and Google earth (Table 1). Cocoons used in this study were collected from *P. bombycina* trees, except those collected from Hahim which were obtained from larvae fed on *P. bombycina* and *L. monopetala*. Cocoons were weighed and placed singly in rearing chambers. Each emergent, fully eclosed moth was given an id tag; its sex, fresh weight, shell weight (after moth eclosion), wing length [44] dimensions recorded. Non- parametric Mann-Whitney U tests were performed using SPSS version 14.0 (IBM Inc., USA) to compare all males with all females for moth weight, forewing length, hind wing length, cocoon weight and shell weight. Individuals of each sex from every population were tested for significant interactions by 2-way ANOVA before proceeding with multiple comparisons using the LSD and Tukey’s HSD test for significance at alpha 0.5. Attempts were also made to derive dendrograms using hierarchical clustering with squared Euclidean distance measures for the morphological parameters studied.

### Isolation of DNA from Moths, Primer Design, and PCR Amplifications

High molecular weight, nuclear genomic DNA was isolated from an abdominal half of each moth using a proteinase K, SDS-based nuclei extraction method [45]. The remaining abdominal half was frozen in liquid nitrogen and stored at –20°C. The quality and quantity of the isolated DNA was ascertained by standard spectro-photometry and 0.8% agarose gel electrophoresis followed by staining with ethidium bromide [46]. For designing anchored ISSR primers, intron sequences of *Antheraea* species available in the public domain NCBI sequence database were collated and searched for sequence repeats using the software mREPS [47]. A total of thirty ISSR primers were designed based on predominant di-, tri-and tetra-nucleotide repeats identified by the above search. Repeats that would yield amplicons in the size range of 250 bp to 2 kbp were selected. Each primer was provided with a unique 3′ or 5′ mononucleotide anchor (Table 2). The primers were synthesized at IDT Inc., USA. PCR amplifications were performed with a profile of 40 cycles of DNA denaturation at 94°C for 30 seconds, primer annealing at 42 to 55°C for 30 seconds (depending upon the T_m_ of the primer used, Table S3) and primer extension at 72°C for 1 minute using an ABI 2720 Thermal cycler. The total reaction volume was 25 µl which contained 0.5 U of Taq DNA polymerase in buffer containing 3 mM magnesium chloride provided by the manufacturer, (Promega Inc., USA), 200 µM of each dNTPs, 30 ng/µl of each primer, and 10 ng of genomic DNA from each moth as template. PCR products were electrophoresed on a 1.5% agarose gel and visualized by ethidium bromide staining. PCR amplifications were performed at least in duplicate to ensure repeatability. Eleven SSR primers were synthesized commercially and applied as recommended in Arunkumar et al., 2010 [9]. Amplification profiles comprised of an initial denaturation at 94°C for 3 minutes followed by 35 cycles of DNA denaturation at 94°C for 20 seconds, primer annealing at recommended temperatures for 10 seconds, primer extension at 72°C for 45 seconds and a final extension at 72°C for 10 minutes using an ABI 2720 Thermo-cycler. Total reaction volume was 10 µl with 0.5 U of *Taq* DNA polymerase in buffer containing 1.5 mM magnesium chloride provided by the manufacturer (Promega Inc., USA), 100 µM of each dNTPs, 5 pmol of each primer, and approximately 10 ng of genomic DNA from each moth as template. All reagents were of molecular biology grade and purchased from Sigma Aldrich Inc., USA, except for the primers, which were synthesized by IDT Inc., USA.

### Population Genetic Analyses of A. assamensis DNAs

Amplification products obtained consistently with each ISSR primer for each insect representing the fifteen populations were scored for the presence (1) or absence (0) of bands. Only repeatable bands in the range of 0.25 to 2.5 kbp were scored. Rare alleles were pruned from the dataset [8]. The number of polymorphic loci and percentage polymorphic loci (P) was determined for each primer at single- and multi-population level. Appropriate estimates of Nei’s gene diversity (H_i_) were obtained using two free-wares: POPGENE version 1.32 and TFPGA version 1.3 [48], [49]. Estimations were done assuming Hardy Weinberg equilibrium conditions (Inbreeding coefficient, F_IS_ = 0), moderate (F_IS_ = 0.5) and high disequilibrium (F_IS_>0.5). A Bayesian approach for estimating total average panmictic heterozygosity (H_S_) was determined reiteratively using Hickory version 1.03 [50] with the f full, f = 0, theta = 0, f-free models and default sampling parameters (Burn-in = 50,000, sample = 250,000 and thin = 50) with three runs. As the deviation information criteria (DIC) were low and statistically similar for f full, f = 0 and f free models, only those values obtained with the f free model are described here. The use of the f free model is touted to be particularly suited for data obtained using dominant markers [51]. SSR-PCR products were manually scored for the presence and size of amplicons. Data from only those individuals for which all three loci could be consistently scored were used. Calculations for observed number of alleles (Na); effective number of alleles (Ne); numbers of haplotypes; estimates of observed, expected and average heterozygosity estimates (H_O_, H_E_ and H_T_) and pair-wise tests for linkage disequilibrium [52] were performed with the SSR-PCR data using Arlequin 3.5 [53] and default parameters.

Data obtained was also converted into haplotype frequencies by AMOVA PREP [54] and CONVERT [55]. Total genetic variation (*Ф*-statistics) was estimated within populations and among populations using by AMOVA [53], [56] using Arlequin 3.1 [53]. Patterns of genetic variation among and within populations comprising groups based on source (wild or cultivated) and region (Upper Assam, Lower Assam and Shillong plateau) were compared. Two groups of cultivated populations from Upper Assam and Lower Assam were also compared. Matrices of inter-population geographic distances (in km) and Nei’s genetic distances estimated from the ISSR data were used to perform a Mantel test using TFPGA and 999 randomized permutations to determine statistical significance [57]. Principal coordinate (PCO) analysis was conducted with Euclidean distances between all ISSR fingerprints of insects from wild and cultivated populations from different regions, using MVSP ver. 3.1 [58]. To detect population structure and genetic admixtures by a Bayesian approach, STRUCTURE 2.3.4. [59], [60] was used with data from ISSR markers and SSR markers. Independent runs at multiple K values (K is the number of inferred clusters) were used for the ISSR data; Multiple independent runs at K = 1 up to K = 15 were attempted for the SSR data. Settings used were burn-in period = 10^5^; no. of MCMC reps after burn-in = 10^5^; Ancestry model with and without admixture; and correlated allele frequency between populations was assumed. Most likely value for assigning K clusters was determined from an *ad-hoc* statistic (ΔK) derived from second order rate of change in likelihood function was used derived from second order rate of change with respect to K of the likelihood function [61]. Where applicable in the above estimations, standard assumptions made for dominant marker data were adopted. These included assuming that each shared ISSR band represented homologous loci and absence of bands could be attributed to lack of primer binding sites in the DNA of some individuals of the populations tested due to chromosomal rearrangements, transposition events and mutations or deletions of pertinent repeat sequences.

### Cloning, Sequencing and Analyses of Selected ISSR-PCR Amplicons

Selected amplicons obtained from individual moths of various populations (Table 2) were gel purified and cloned into the pDrive vector according to the manufacturer’s instructions (Qiagen Inc., USA). Reasons for selecting these amplicons for sequencing are described in the Results section. Plasmid DNA was isolated from positive colonies using Qiagen plasmid purification kit, and sequenced on both strands at Macrogen Inc., South Korea. At least three clones were sequenced to confirm each sequence. The sequences were examined with an online software Spectral Repeat Finder or SRF [62] which uses Fourier transformation to search sequences for repeat patterns, regions of repeats in a sequence, copy number and percentage nucleotide content. The sequences were also analyzed for the presence of tandem repeats using mREPS [47]. The sequences were used to search the annotated nucleic acid databases at GenBank using BLAST-n [63], and the repeat libraries using RepeatMasker [64] and Censor [65] using default parameters. Sequences have been submitted to the NCBI database as accessions DQ872512, DQ872513, DQ872514, EU123527, EU123528, EU123529, EU123530, GQ273748, GQ273749, GQ273750, GQ273751, GQ273752, GQ273753, GQ273754, GQ273755, GQ273756 and GQ273757.

### Southern Hybridizations Using an Amplicon Containing a Repetitive Element as Probe

Southern hybridizations were performed after Mazumdar-Leighton and Broadway [66]. High molecular weight nuclear genomic DNA isolated from single moths of *A. assamensis* (BK-12 from LA region, TR-3 from SP, TR-4 from SP, KG-8 from UA), *B. mori, S. cynthia A. proylei* and *A. mylitta* was digested completely with the restriction enzyme *Hin*d III and *Hae* III (New England Biolabs, Inc., MA, USA). The probe used was an 800 bp amplicon obtained by nested PCR (using primers Fp2: 5′CTATGTCATCCACTGTACTGTAATT3’ and Ah16: 5′TTTCACGAACCCATACA GGC3’) from a plasmid clone DQ872512 containing a polymorphic locus TR4_1456P_ obtained with primer AhISSR110. This locus occurred in the dataset with a frequency of 37% and showed high sequence similarity to lepidopteran transposable elements. The 800 bp amplicon contained an internal restriction site for *Hae* III but lacked sites for *Hin*d III. Low stringency washes (4X SSC +0.1%SDS) were done twice for 30 minutes at 65°C before overnight autoradiography at −80°C. Subsequent high stringency washes (2XSSC +0.1%SDS, 0.5X SSC +0.1%SDS) for 30 minutes each at 65°C was followed by 2 days autoradiography at −80°C. Primers Fp2 and Ah16 used for nested PCR and amplification of the probe were also used to amplify DNA from the heterologous insects, *B. mori, S. cynthia, A. proylei* and *A. mylitta*. These PCRs were performed as described earlier except that a primer annealing temperature of 60°C was used.

## Supporting Information

Figure S1 **A tree depicting clusters among 15 populations of** ***A. assamensis*** **based on Nei’s genetic distances.** The number of loci supporting each node and proportion of similar replicates (bootstrap) calculated using TFPGA is indicated in parentheses. Population color codes are the same as Figure 2A.
(TIF)

Figure S2 **Mean (±SD) weight of moth weight (MW), cocoon weight (CW) and shell weight (SW) of male (M) □ and female (F) ▪** ***A. assamensis*** **.**
(TIF)

Figure S3 **Relation between moth weight and cocoon weight in male (Δ) and female ( )** ***A. assamensis.*** The r^2^ values are also indicated.
(TIF)

Table S1 **Partitioning of genetic variation for the full set of** ***A. assamensis*** **moths using (A) ISSR marker data and (B) SSR marker data.** *Significance tests were performed at 1023 permutations. **Bayesian analogue (θii value) was 0.32+0.02.
(DOC)

Table S2 **Matrix showing pair-wise estimates of Nei’s genetic distance estimates based on 50 loci from ISSR data.** Wild populations from Shillong plateau are shown in bold, cultivated populations from Lower Assam region are italicized. Populations showing the highest and lowest genetic distance estimates are italicized in bold.
(DOC)

Table S3 **Details of thirty ISSR primers designed from intronic repeats within Antheraea genes and their ability to amplify lepidopteran genomic DNAs.**
(DOC)
