# Supplementary figures and images for: Genetic Variation within Native Populations of Endemic Silkmoth Antheraea assamensis (Helfer) from Northeast India Indicates Need for In Situ Conservation
Source: PLoS One. 2012 Nov 21;7(11):e49972. doi: 10.1371/journal.pone.0049972 (PMC3503872; doi:10.1371/journal.pone.0049972)

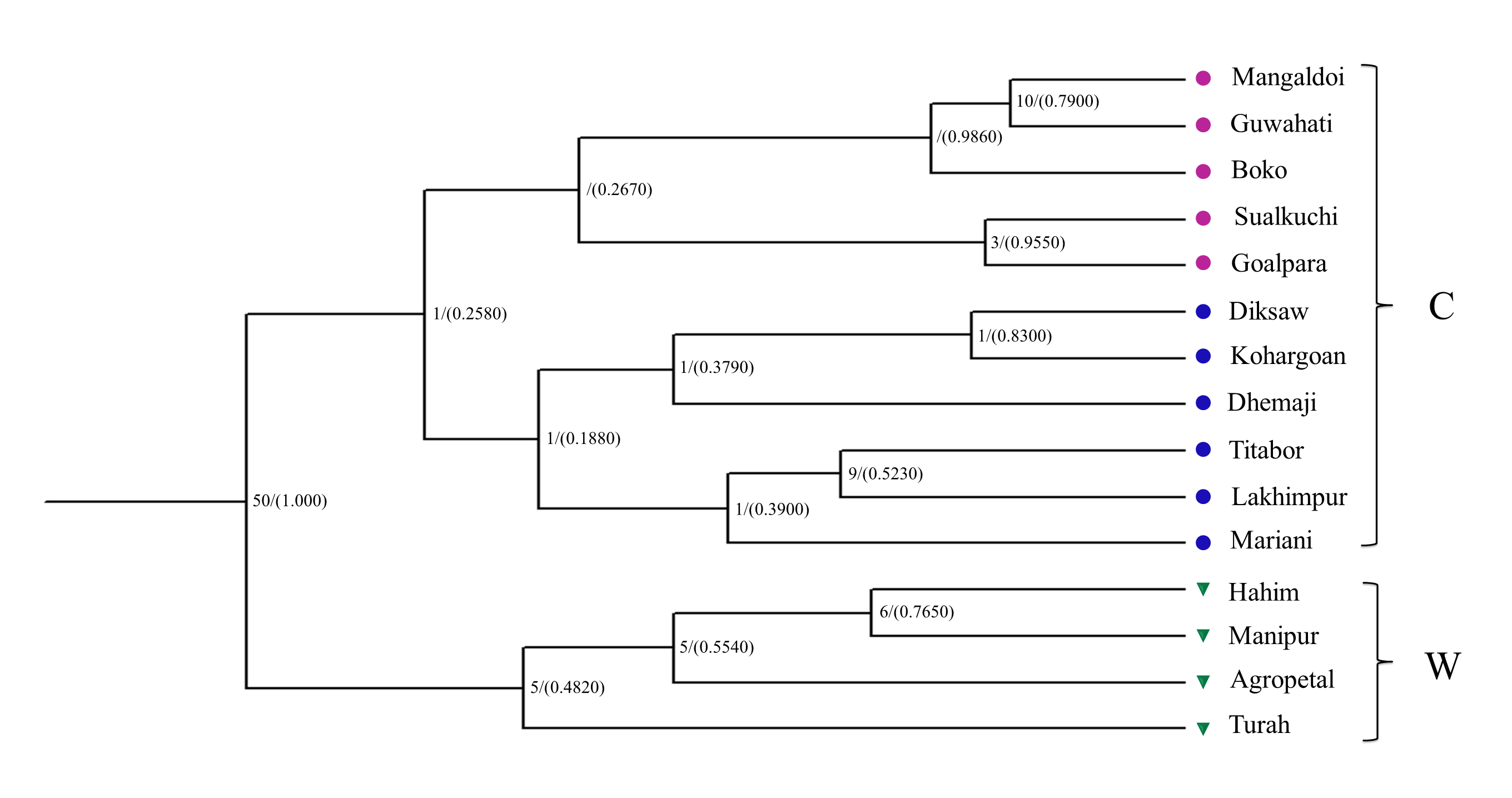

Supplement: Figure S1 — A tree depicting clusters among 15 populations of A. assamensis based on Nei’s genetic distances. The number of loci supporting each node and proportion of similar replicates (bootstrap) calculated using TFPGA is indicated in parentheses. Population color codes are the same as Figure 2A. (TIF) [file pone.0049972.s001.tif]

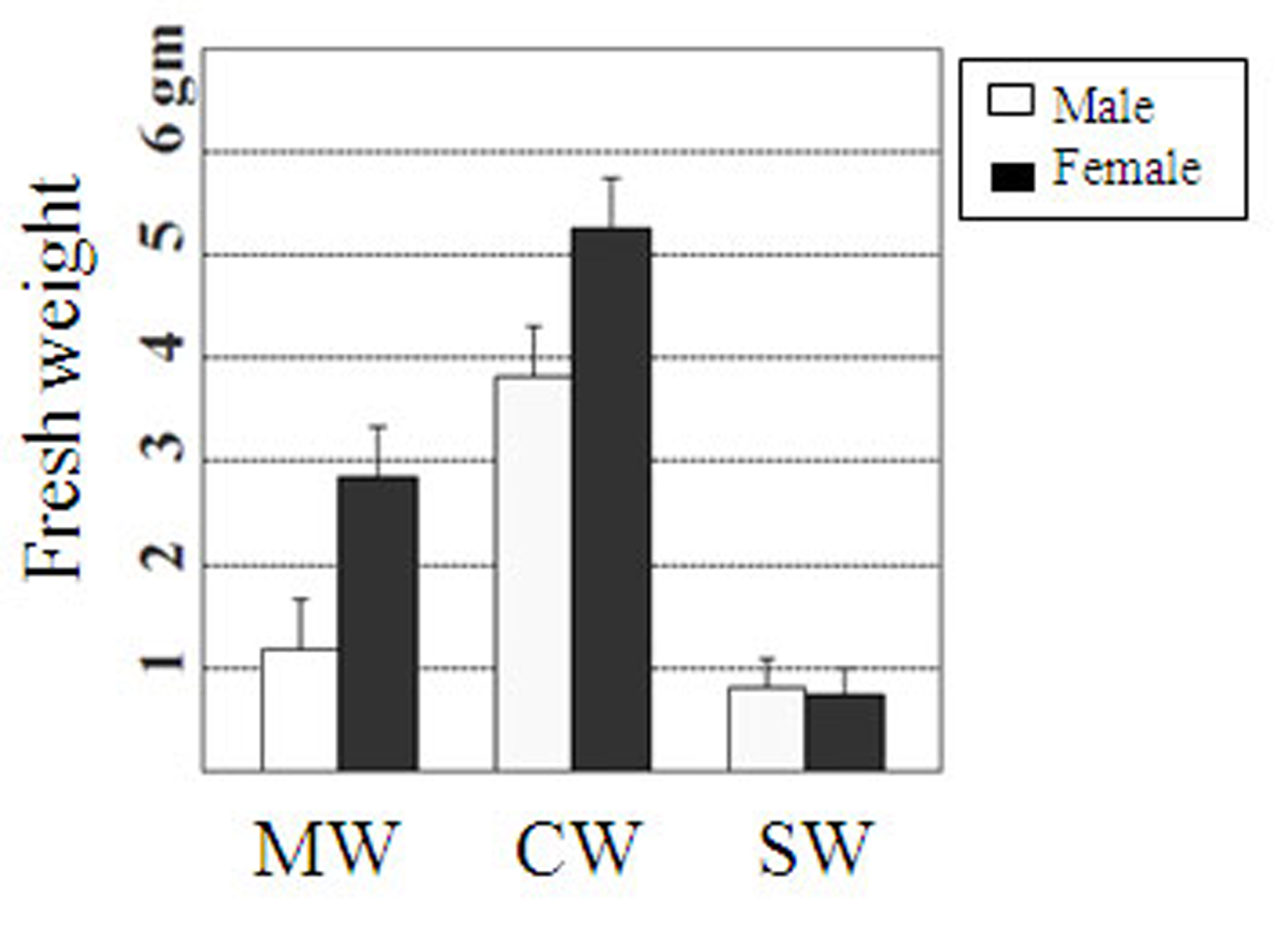

Supplement: Figure S2 — Mean (±SD) weight of moth weight (MW), cocoon weight (CW) and shell weight (SW) of male (M) □ and female (F) ▪ A. assamensis . (TIF) [file pone.0049972.s002.tif]

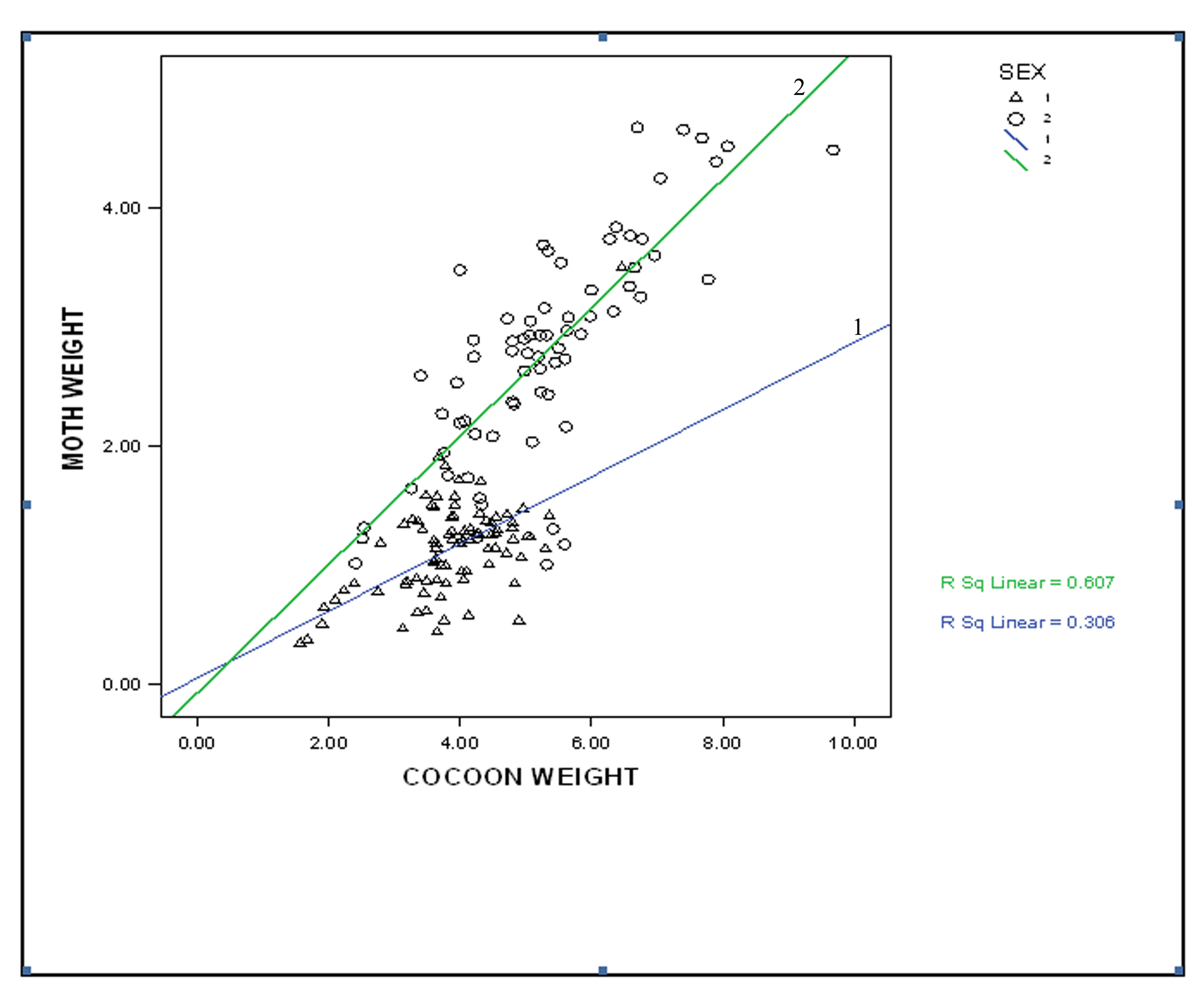

Supplement: Figure S3 — Relation between moth weight and cocoon weight in male (Δ) and female ( ) A. assamensis. The r2 values are also indicated. (TIF) [file pone.0049972.s003.tif]
