# Supplementary material for: Genetic Variation within Native Populations of Endemic Silkmoth Antheraea assamensis (Helfer) from Northeast India Indicates Need for In Situ Conservation
Source: PLoS One. 2012 Nov 21;7(11):e49972. doi: 10.1371/journal.pone.0049972 (PMC3503872; doi:10.1371/journal.pone.0049972)
Supplement: Table S1 — Partitioning of genetic variation for the full set of A. assamensis moths using (A) ISSR marker data and (B) SSR marker data. *Significance tests were performed at 1023 permutations. **Bayesian analogue (θii value) was 0.32+0.02. (DOC) [file pone.0049972.s004.doc]

Table S1: Partitioning of genetic variation for the full set of *A. assamensis* moths using (A) ISSR marker data and (B) SSR marker data. *Significance tests were performed at 1023 permutations. **Bayesian analogue (Ɵ_ii_ value) was 0.32+0.02.

|  | **Source of variation** | **d. f.** | **Variance components** | **Percentage of variation** | **Fixation indices** | **P-value*** |
| --- | --- | --- | --- | --- | --- | --- |
| **A.** | Among populations | 14 | 2.87 | 35.86 | F_ST_=0.36** | 0 |
|  | Within populations | 192 | 5.12 | 64.14 |  |  |
|  | Total | 206 | 7.99 |  |  |  |
| **B.** | Among populations | 14 | 0.08 | 18.33 | F_ST_=0.18 | 0 |
|  | Within populations | 363 | 0.37 | 81.67 |  |  |
|  | Total | 377 | 0.46 |  |  |  |
