# Supplementary material for: Genetic Variation within Native Populations of Endemic Silkmoth Antheraea assamensis (Helfer) from Northeast India Indicates Need for In Situ Conservation
Source: PLoS One. 2012 Nov 21;7(11):e49972. doi: 10.1371/journal.pone.0049972 (PMC3503872; doi:10.1371/journal.pone.0049972)
Supplement: Table S2 — Matrix showing pair-wise estimates of Nei’s genetic distance estimates based on 50 loci from ISSR data. Wild populations from Shillong plateau are shown in bold, cultivated populations from Lower Assam region are italicized. Populations showing the highest and lowest genetic distance estimates are italicized in bold. (DOC) [file pone.0049972.s005.doc]

Table S2: Matrix showing pair-wise estimates of Nei’s genetic distance estimates based on 50 loci from ISSR data. Wild populations from Shillong plateau are shown in bold, cultivated populations from Lower Assam region are italicized. Populations showing the highest and lowest genetic distance estimates are italicized in bold.

| **Pop ID** | **BK** | **MD** | **NB** | **MR** | **SK** | **GP** | **DM** | **D** | **KG** | **TB** | **LK** | **H** | **MN** | **TR** | **AG** |
| --- | --- | --- | --- | --- | --- | --- | --- | --- | --- | --- | --- | --- | --- | --- | --- |
| **BK** | ****** |  |  |  |  |  |  |  |  |  |  |  |  |  |  |
| **MD** | *0.0595* | ****** |  |  |  |  |  |  |  |  |  |  |  |  |  |
| **NB** | *0.0739* | ***0.0457*** | ****** |  |  |  |  |  |  |  |  |  |  |  |  |
| **MR** | *0.1482* | *0.1708* | *0.1849* | ****** |  |  |  |  |  |  |  |  |  |  |  |
| **SK** | *0.1985* | *0.1485* | *0.1281* | *0.2469* | ****** |  |  |  |  |  |  |  |  |  |  |
| **GP** | *0.2006* | *0.1494* | *0.1303* | *0.1872* | *0.0523* | ****** |  |  |  |  |  |  |  |  |  |
| **DM** | 0.1709 | 0.1631 | 0.1583 | 0.1556 | 0.2645 | 0.2303 | **** |  |  |  |  |  |  |  |  |
| **D** | 0.1503 | 0.1693 | 0.1658 | 0.1074 | 0.1968 | 0.1955 | 0.1034 | **** |  |  |  |  |  |  |  |
| **KG** | 0.1644 | 0.1800 | 0.1702 | 0.1635 | 0.2072 | 0.2515 | 0.1657 | 0.0559 | **** |  |  |  |  |  |  |
| **TB** | 0.2652 | 0.2473 | 0.2388 | 0.1102 | 0.1771 | 0.1178 | 0.1730 | 0.0891 | 0.1787 | **** |  |  |  |  |  |
| **LK** | 0.2564 | 0.2662 | 0.2640 | 0.1299 | 0.2192 | 0.1709 | 0.2322 | 0.1595 | 0.2709 | 0.0906 | **** |  |  |  |  |
| **H** | **0.1766** | **0.2076** | **0.1957** | **0.2358** | **0.2628** | **0.2388** | **0.2404** | **0.2335** | **0.2750** | **0.2821** | **0.1717** | ******** |  |  |  |
| **MN** | **0.2085** | **0.2382** | **0.1854** | **0.3167** | **0.1860** | **0.1889** | **0.2495** | **0.2898** | **0.3254** | **0.2892** | **0.1980** | **0.1550** | ******** |  |  |
| **TR** | **0.1839** | **0.1738** | **0.1705** | **0.3003** | **0.2001** | **0.1896** | **0.2692** | **0.2743** | **0.3011** | **0.3164** | **0.2820** | **0.1975** | **0.1434** | ******** |  |
| **AG** | **0.1410** | **0.1737** | **0.1735** | **0.3263** | **0.2765** | **0.2875** | **0.2634** | **0.2779** | **0.2790** | ***0.3916*** | **0.2981** | **0.1038** | **0.1886** | **0.1849** | ******** |
