# Supplementary material for: Genetic Variation within Native Populations of Endemic Silkmoth Antheraea assamensis (Helfer) from Northeast India Indicates Need for In Situ Conservation
Source: PLoS One. 2012 Nov 21;7(11):e49972. doi: 10.1371/journal.pone.0049972 (PMC3503872; doi:10.1371/journal.pone.0049972)
Supplement: Table S3 — Details of thirty ISSR primers designed from intronic repeats within Antheraea genes and their ability to amplify lepidopteran genomic DNAs. (DOC) [file pone.0049972.s006.doc]

Table S3: Details of thirty ISSR primers designed from intronic repeats within *Antheraea* genes and their ability to amplify lepidopteran genomic DNAs.

| **S. No** | **Primer Name** | **Primer Sequence** | **%GC** | **T_m_ (^0^C)** | **Amplification of lepidopteran DNA*** |
| --- | --- | --- | --- | --- | --- |
| 1 | AhISSR 1 | (AAT)_6_C | 5.26 | 36.7 | - |
| 2 | AhISSR 2 | (GAA)_6_C | 36.84 | 49.2 | Aa, Ai, Am, Ap, Bm, Sc |
| 3 | AhISSR 3 | (AATT)_5_C | 4.76 | 44 | - |
| 4 | AhISSR 4 | (TCG) _6_C | 68.42 | 73.6 | Aa, Ai, Am, Ap, Bm, Sc |
| 5 | AhISSR 5 | (CGA) _6_T | 63.17 | 70 | Aa, Ai, Am, Ap, Bm, Sc |
| 6 | AhISSR 6 | (ATTC) _5_C | 28.57 | 57.7 | Bm |
| 7 | AhISSR 7 | (AGCT) _5_ | 52.38 | 56 | - |
| 8 | AhISSR 8 | (TACT) _5_C | 28.57 | 41.4 | Aa, Ai, Am, Ap, Bm, Sc |
| 9 | AhISSR 9 | (ATAC) _5_C | 28.57 | 45.1 | Aa, Ai, Am, Ap, Bm, Sc |
| 10 | AhISSR 10 | (TTA) _6_C | 5.26 | 35.2 | - |
| 11 | AhISSR 11 | C(GATA) _5_ | 28.57 | 44.4 | Aa, Ai, Am, Ap, Bm, Sc |
| 12 | AhISSR 12 | (GATA) _5_T | 23.8 | 39.7 | Aa, Ai, Am, Ap, Bm, Sc |
| 13 | AhISSR 13 | (GATA) _5_A | 23.8 | 40.8 | Aa, Ai, Am, Ap, Bm, Sc |
| 14 | AhISSR 14 | (GCCGGTGCTGT)2 | 75 | 84.7 | - |
| 15 | AhISSR 120 | (TTA) _6_TG | 5.26 | 32.7 | **-** |
| 16 | AhISSR 103 | (GT)_9_C | 52.63 | 53.8 | Aa, Ai, Am, Ap, Bm, Sc |
| 17 | AhISSR 110 | (GATA) _5_C | 28.57 | 40 | Aa, Ai, Am, Ap, Bm, Sc |
| 18 | AhISSR 18 | (GGCC) _5_C | 100 | 80.7 | **-** |
| 19 | AhISSR 19 | C(GAA)_6_ | 36.84 | 46.7 | **-** |
| 20 | AhISSR 20 | C(TCG)_6_ | 68.42 | 60.2 | Aa, Ai, Am, Ap, Bm, Sc |
| 21 | AhISSR 21 | T(CGA)_6_ | 63.16 | 60.6 | Aa, Ai, Am, Ap, Bm, Sc |
| 22 | AhISSR 22 | T(GATA)_5_ | 23.81 | 40.7 | Aa, Ai, Am, Ap, Bm, Sc |
| 23 | AhISSR 23 | G(GATA)_5_ | 23.81 | 40.4 | Aa, Ai, Am, Ap, Bm, Sc |
| 24 | AhISSR 24 | (GACA)_5_C | 52.38 | 54.8 | Aa, Ai, Am, Ap, Bm, Sc |
| 25 | AhISSR 25 | (CA)_10_T | 47.62 | 56 | Aa, Ai, Am, Ap, Bm, Sc |
| 26 | Ayvit 1 | (CAAA)_5_T | 23.81 | 46.6 | Aa, Ai, Am, Ap, Bm, Sc |
| 27 | Ayvit 2 | (TAA)_6_C | 5.26 | 30.5 | - |
| 28 | Ayvit 3 | (CT)_10_A | 47.62 | 48.1 | Aa, Ai, Am, Ap, Bm, Sc |
| 29 | Ayvit 4 | (ATG)_6_C | 36.84 | 47.4 | Aa, Ai, Am, Ap, Bm, Sc |
| 30 | Ayvit 5 | (TA)_10_G | 4.76 | 26.7 | - |

* Aa: *Antheraea assamensis,* Ai: *Agrotis ipsilon*, Am: *Antheraea mylitta,*Ap: *Antheraea proylii,* Bm: *B.mori,* Sc: *Samia cynthia*; (-) refers to non-amplification.
